# In Vitro Regeneration Potential of White Lupin *(Lupinus albus*) from Cotyledonary Nodes

**DOI:** 10.3390/plants9030318

**Published:** 2020-03-03

**Authors:** Mehtab Muhammad Aslam, Joseph K. Karanja, Qian Zhang, Huifeng Lin, Tianyu Xia, Kashif Akhtar, Jianping Liu, Rui Miao, Feiyun Xu, Weifeng Xu

**Affiliations:** 1 Center for Plant Water-Use and Nutrition Regulation, College of Life Sciences, Joint International Research Laboratory of Water and Nutrient in Cops, Fujian Agriculture and Forestry University, Fuzhou, Fujian 350002, China; 2171916002@fafu.edu.cn (M.M.A.); 2171916001@fafu.edu.cn (J.K.K.); 2170525005@fafu.edu.cn (H.L.); tyxia@fafu.edu.cn (T.X.); jpliu@fafu.edu.cn (J.L.); ruimiao@fafu.edu.cn (R.M.); 2181902002@fafu.edu.cn (F.X.); 2 Sanming Academy of Agriculture Sciences, Sanming, Fujian 350002, China; 3 Institute of Nuclear Agricultural Sciences, Key Laboratory of Nuclear Agricultural Sciences of Ministry of Agriculture and Zhejiang Province, Zhejiang University, Hangzhou 310058, China; kashif@zju.edu.cn

**Keywords:** plant regeneration, shoot propagation, auxin, cytokinin, carbohydrate source, white lupin

## Abstract

The tissue culture regeneration system of *Lupinus albus* has always been considered as recalcitrant material due to its genotype-dependent response and low regeneration efficiency that hamper the use of genetic engineering. Establishment of repeatable plant regeneration protocol is a prerequisite tool for successful application of genetic engineering. This aim of this study was to develop standardized, efficient protocol for successful shoot induction from cotyledonary node of white lupin. In this study, 5 day old aseptically cultured seedlings were used to prepare three explants (half cotyledonary node, HCN; whole cotyledonary node, WCN; and traditional cotyledonary node, TCN), cultured on four concentrations of M519 medium (M519, ½ M519, ^1/3^ M519, and ¼ M519), containing four carbohydrate sources (sucrose, fructose, maltose, and glucose), and stimulated with various combinations of KT (kinetin), and NAA (naphthalene acetic acid) for direct shoot regeneration. High frequency of 80% shoot regeneration was obtained on ½ M519 medium (KT 4.0 mg L^−1^ + NAA 0.1 mg L^−1^) by using HCN as an explant. Interestingly, combinations of (KT 4.0 mg L^−1^ + NAA 0.1 mg L^−1^ + BAP 1.67 mg L^−1^), and (KT 2.0 mg L^−1^ + NAA 0.1 mg L^−1^) showed similar shoot regeneration frequency of 60%. Augmentation of 0.25 g L^−1^ activated charcoal (AC) not only reduced browning effect but also improved shoot elongation. Among the all carbohydrate sources, sucrose showed the highest regeneration frequency with HCN. Additionally, 80% rooting frequency was recorded on ½ M519 containing IAA 1.0 mg L^−1^ + KT 0.1 mg L^−1^ (indole acetic acid) after 28 days of culturing. The present study describes establishment of an efficient and successful protocol for direct plant regeneration of white lupin from different cotyledonary nodes.

## 1. Introduction

White lupin *(Lupinus albus L.)* is an important legume plant and produces high protein content seeds, dietary fiber, starch or gluten free, [1] and have good nutritional value [1,2]. Besides its nutritional importance, white lupin, is capable of fixing atmospheric N_2_ into accessible form for plants [3]. White lupin is also a cluster root (CR) forming legume and illuminated as model plant to examine morphological and biochemical adaptations of plants to phosphate deficient soils [4], and plays a key role in improving soil exploration and phosphate availability [5,6,7].

Phosphate (P) is major limiting macronutrient which is depleted at an alarming rate due the fact of its limited reserves [8,9], CR (short densely formed lateral roots) appeared as an important trait in improving fertilizer use efficiency and nutrition acquisition [10]. Therefore, white lupin has generated heightened interest among researchers aiming to generate P-efficient genetically modified improved plants, due to the fact of its versatile application in agriculture. However, most of the legume species [11,12], especially white lupin, are considered as recalcitrant material for genetic manipulation. 

Highly efficient and reliable shoot regeneration systems are essential for propagation of in vitro genetic manipulation [13] e.g., production of transgenic plants [14]. Plant tissue culture system is an efficient in vitro based technique used for genetic modification and plant regeneration, and reduces yield loss [15,16,17]. Development of plants with potential improved traits such as superior quality, high yield, better adaptability, disease tolerance and stress resistance is mainly based on micropropagation technique of tissue culture system [18,19,20]. Relatively very poor progress has been accomplished on plant regeneration system in legumes [21], due to the unsuccessful in vitro generation tissue culture systems. Several attempts have previously been made to develop successful regeneration system in legumes such as *Glycine max* [22], *Vigna unguiculata* [23], *Medicago sativa* [24], *Phaseolus vulgaris* [25] *Arachis hypogaea* [26] *Trifolium resupinatum* [27], *Lathyrus sativus* [28], *Vigna aconitifolia* [29], *Onobrychis viciifolia* [30], and *Stylosanthes humilis* [31], *Lupinus angustifolius* [32], *L. luteus* [32,33,34], and *L. albus* [32,35]. Uhde-Stone et al, demonstrated successful transgenic proteoid root development of white lupin by using *Agrobacterium rhizogenes* [36]. To the best of our knowledge, very limited studies are available on in vitro shoot multiplication of white lupin. 

Several factors can influence shoot multiplication [16] such as selection of explant, media type [37], concentration of plant growth regulators, and carbon energy source [38] which maintains osmotic potential [39]. Carbon source is an essential supplement of tissue culture medium that regulates photosynthetic activity of explants. Generally, sucrose is the most preferable carbon source for generation of multiple shoots via tissue culture system due to its critical role in facilitating plant growth and differentiation [40,41]. Few researchers have suggested different types of carbon sources that may have distinct effect on tissue morphogenesis and plant growth [38,42,43], and evaluation of their individual effects on each plant species is necessary. Regeneration protocol of each cultivar is widely different from other cultivars even within the same species [44]. 

Plant regeneration success is mainly dependent on nature of the explant, type of medium, cultivar, carbohydrate source, and combination of plant hormones [45]. However, there is an urgent need to establish efficient regeneration protocol for each plant species [46]. An efficient regeneration protocol is a pre-requisite tool for high frequency shoot regeneration and development of transgenic white lupin plant with improved potential traits. The aim of this research effort was to develop successful shoot regeneration protocol in white lupin by using three cotyledonary nodes. A new efficient protocol with high frequency of in vitro shoot regeneration was established using different concentrations of plant growth regulators and activated charcoal on ½ M519 media. Furthermore, the effect of explant, medium, and carbon sources was optimized to evaluate the best shoot regeneration frequency. This optimized regeneration protocol will provide future direction for the development of genetically modified transgenic white lupin and other legume crops. 

## 2. Results

### 2.1. Efficiency of Shoot Regeneration from Three Cotyledonary Nodes 

**Figure 1:**
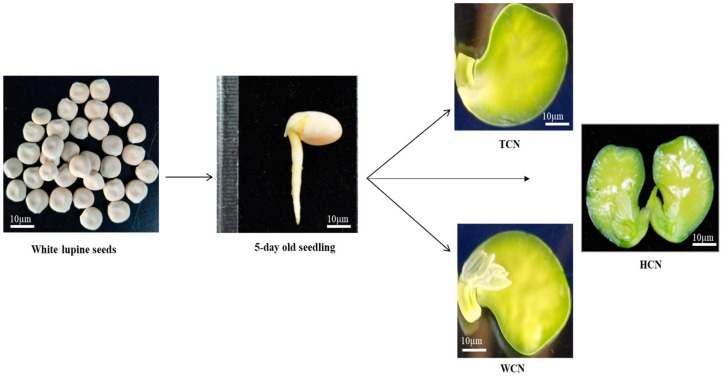
Preparation of explants from 5 day old seedlings of *Lupinus albus*. TCN (traditional cotyledonary node), HCN (half cotyledonary node), and WCN (whole cotyledonary node).

**Figure 2:**
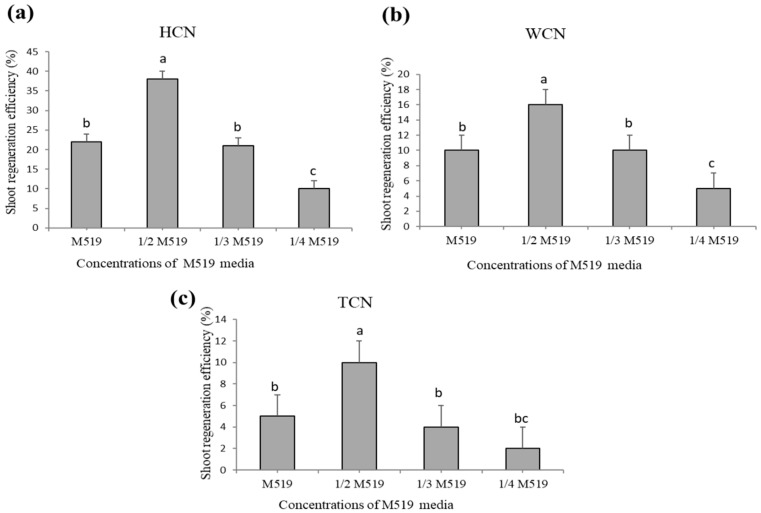
Shoot regeneration efficiency of *Lupinus albus* from three different explants on four different concentrations of basal media. (**a**) HCN (half cotyledonary node), (**b**) WCN (whole cotyledonary node), (**c**) TCN (traditional cotyledonary node). HCN showed highest regeneration frequency and higher number of shoots on ½ M519 medium supplemented with (KT 4.0 mg L^−1^, NAA 0.1 mg L^−1^), than WCN and TCN. Different letters show significant differences at *p* < 0.05. Data shown represent mean ± standard error of four replicates.

Different explants from various cotyledons: HCN (half cotyledonary node), WCN (whole cotyledonary node), and TCN (traditional cotyledonary node) are shown below in Figure 1. Half cotyledonary node showed highest regeneration frequency (80%) (Figure 2a) and higher number of shoots explant^−1^ (16 explant^−1^) on ½ M519 medium supplemented with (KT 4.0 mg L^−1^, NAA 0.1 mg L^−1^) (Table 1). All treatments with HCN explants resulted in higher shoot regeneration frequency than the other two explants. WCN showed intermediate shoot regeneration frequencies under same conditions (Figure 2b), while TCN exhibited very poor regeneration frequency (Figure 2c). Significant differences were observed among the three types of explants. Although no significant differences were found on shoot regeneration by germinating seeds on ½ MS medium or sterilized ddH_2_O. 

### 2.2. Concentration of M519 Medium-Affected Shoot Regeneration 

Different formulations of M519 medium (M519, ½ M519, ^1/3^ M519, ¼ M519) were used to investigate its effect on shoot regeneration rate by using HCN (Figure 2a), WCN (Figure 2b), and TCN (Figure 2c) explants. Interestingly, the same medium with different ratios showed different response on the ability of shoot regeneration, whereby maximum shoot regeneration was obtained on only ½ M519 (half strength media). However, no significant differences were observed among full M519, and ^1/3^ M519 medium, while ¼ M519 showed significantly lower regeneration rate relative to all other media concentrations (Figure 2a–c). Therefore, it can be inferred that the basal medium selected for shoot regeneration can greatly affect plant regeneration ability. Transferring explants after every two weeks on same medium has reduced shoot tip necrosis and enhanced shoot proliferation. This data was collected after 4 to 5 weeks of initial culturing of explant. 

### 2.3. Effect of Carbohydrate Source on Shoot Regeneration

**Figure 3:**
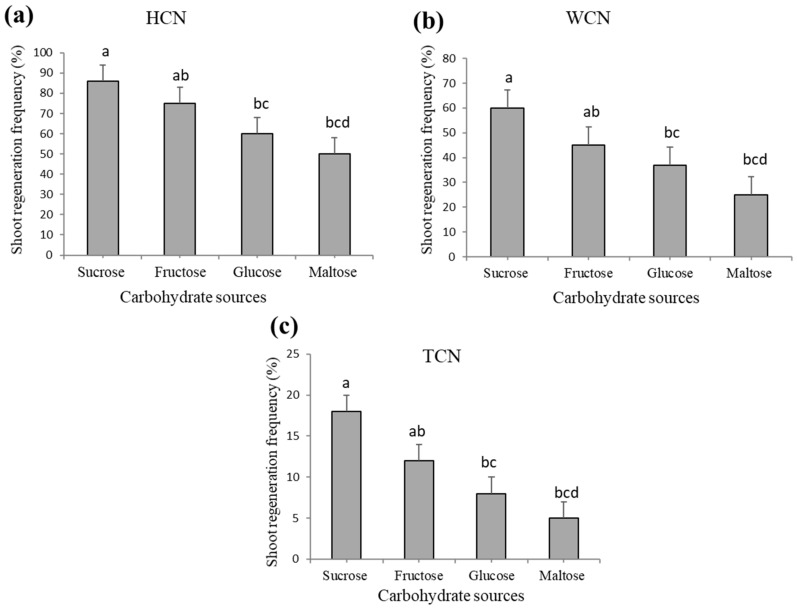
Shoot regeneration efficiency of *Lupinus albus* from three different explants on four different carbohydrate sources after 4 to 5 weeks of culturing (**a**) HCN (half cotyledonary node), (**b**) WCN (whole cotyledonary node), (**c**) TCN (traditional cotyledonary node). Different letters show significant differences at *p* < 0.05. Data shown represent mean ± standard error of four replicates.

Sucrose is the most likely used carbohydrate source due to its low cost and availability. In this study four different carbon source (i.e., sucrose, fructose, glucose, and maltose) were used on same M519 medium. Three percent sucrose was found to induce the highest shoot regeneration frequency than all other carbohydrate sources in all the three explants. A decreasing tread of regeneration frequency was observed in the order of sucrose > fructose > glucose > maltose from the three explants (Figure 3a–c), different letters indicates significant differences were found among four carbohydrate sources. These data were collected after 4 to 5 weeks of explant culturing on different carbohydrate supplemented media. 

### 2.4. Shoot Regeneration and Propagation

The data were recorded after 4 to 5 weeks, direct shoot regeneration of white lupin was achieved by using three explants on ½ M519 medium fortified with 3% sucrose, 0.5 g L^−1^ MES, and various combinations of plant growth regulators. The results revealed that, different regeneration pathway affects the potential ability of explants to regenerate. 

**Figure 4:**
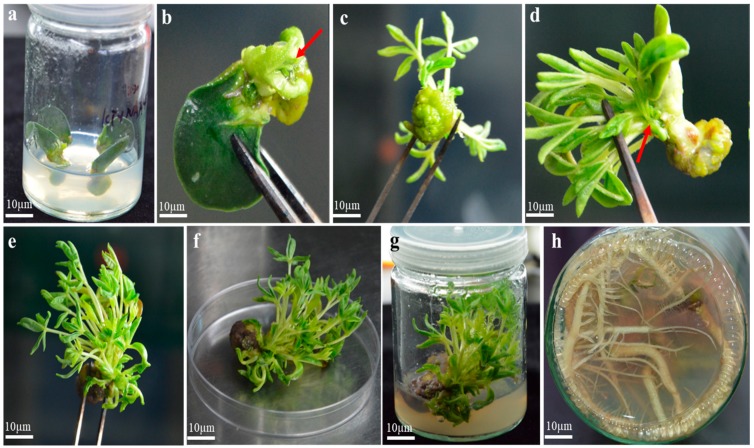
Whole regeneration of *Lupinus albus* on ½ M519 medium using HCN explants. (**a**) HCN inoculated on ½ M519 media (**b**) after 7 days cotyledon develop new bud (**c**) after 14 days small plantlet appears (**d**) after 21 days more shoots generate (**e**) after 28 days shoot length increase (f) after 28 days plant bud turns into dark brown color (**g**) plant inoculated on medium containing (KT 4.0 mg L^−1^ + NAA 0.1 mg L^−1^) (**h**) individual shoot cultured on ½ M519 medium with (IAA 1.0 mg L^−1^ + KT 0.1mg L^−1^).

Eighty percent shoot regeneration frequency was obtained on ½ M519 medium supplemented with 3% sucrose, KT 4.0 mg L^−1^+ NAA 0.1 mg L^−1^ by using HCN, while 60% shoot regeneration was obtained with KT 2.0 mg L^−1^ + NAA 0.1 mg L^−1^ and KT 2.0 mg L^−1^ + NAA 0.1 mg L^−1^ + BAP 1.67 mg L^−1^. Other combinations showed relatively low regeneration frequency (Table 1). After successful regeneration, shoot data were recorded, bunch of multiple shoots were dissected individually on the same medium with 0.25 g L^−1^ activated charcoal, and dead cotyledons were separated (Figure 4a–g).

**Figure 5:**
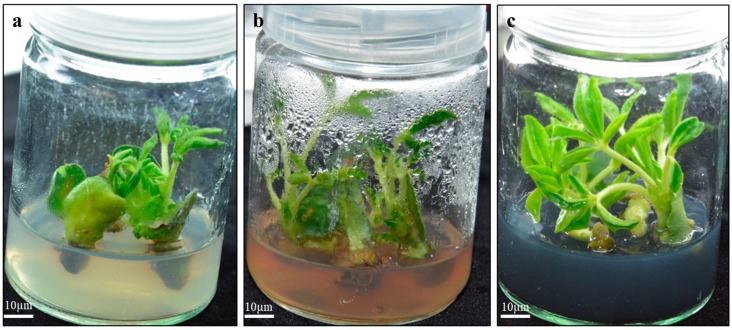
Effect of phenolic secretion on regeneration efficiency of explants. (**a**) HCN cultured on shoot induction medium. (**b**) After 2 weeks of culturing media turning into brown color due to the antioxidant secretion, (**c**) activated charcoal 0.25 g L^−1^ was added into the shoot elongation medium and successfully inhibited tissue browning.

After 2 weeks of culturing, explants turned into brown color from the bottom due the exudation of phenolic compounds in the medium. Activated charcoal acts as an antioxidant to detoxify the effect of phenolic on the regeneration process. To prevent browning effect on shoot regeneration, ½ M519 medium was supplemented with 0.25 g L^−1^ activated charcoal (Figure 5c). 

### 2.5. Root Induction

**Figure 6:**
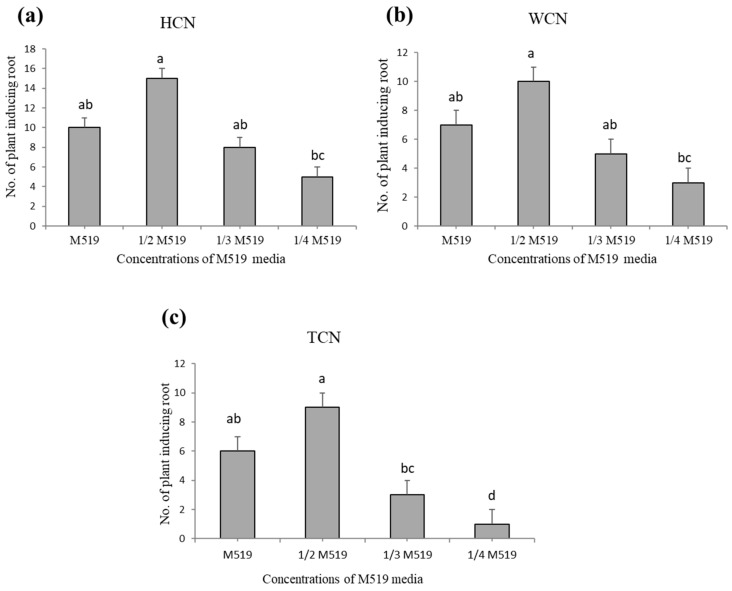
Root induction of *Lupinus albus* from three different explants on four different ratios of M519 media. (**a**) HCN (half cotyledonary node), (**b**) WCN (whole cotyledonary node), (**c**) TCN (traditional cotyledonary node). Different letters show significant differences at *p* < 0.05. Data shown represent mean ± standard error of four replicates.

Elongated shoots of 5–6 cm were separated and cultured on rooting media. After 2 weeks of culture, the rooting initiated directly from the excised portion of the shoots. Shoot generated from HCN, WCN, and TCN were inoculated on different formulations of M519 medium, and their effect of root induction efficiency was determined (Figure 6). The highest percentage of rooting was obtained on ½ M519 medium supplemented with 3% sucrose and IAA 1.0 mg L^−1^ + KT 0.1 mg L^−1^ (Figure 4h), while IAA alone 1.0mg L^−1^ showed (66%) rooting percentage. Interestingly, the root induction was decreased with increasing concentration of IAA. Concentrations of IBA alone showed reduced root induction, while IBA in combination with auxin (IAA 2.0 mg L^−1^ + IBA 0.5 mg L^−1^) showed 60% root induction percentage. The results of all other combinations of IAA, IBA, and KT on rooting percentage and number of roots are shown in Table 2 below. 

### 2.6. Regeneration Cycle of White Lupin 

**Figure 7:**
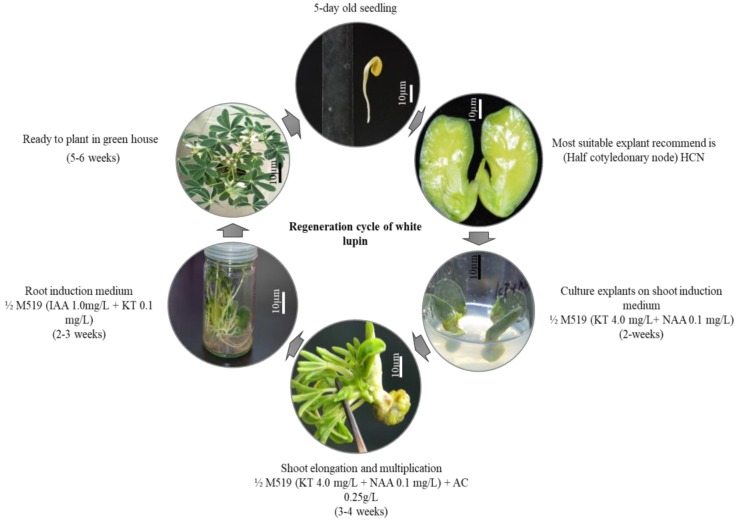
Full regeneration cycle of *Lupinus albus*. Different steps of direct regeneration are shown with their timelines. The regeneration cycle from cotyledons to seed collection takes ~18 weeks. It can be shortened by another two weeks by skipping the shoot elongation step and increasing the shoot initiation medium incubation for three weeks (light arrow) (Shoot initiation medium; shoot elongation medium; root induction medium).

The complete regeneration cycle of white lupin with time scale is shown in Figure 7 below. It is estimated that, the complete regeneration cycle may take 17 to 18 weeks, although it can be shortened by two weeks by skipping shoot elongation step and increasing shoot induction duration for 3 weeks.

## 3. Discussion

The current research was designed to evaluate the regeneration potential of *Lupinus albus* belonging to the family Fabaceae. White lupin (*Lupinus albus*) is considered as recalcitrant material for genetic transformation as well as regeneration process. To date, the regeneration and transformation success of White Lupin has not been achieved. Success of plant regeneration relies on several factors such as selection of genotype, explant nature [47], type of medium, carbon source, phytohormonal combinations, and in vitro regeneration efficiency [48]. Therefore, a key pre-requisite to successful plant regeneration process involves establishment of a standardized regeneration protocol. 

In previous studies, regeneration of few *Lupin spp*. has been described by using different explants such as hypocotyl, petiole and leaf [47,49]. It was further reported that, explant type and age can significantly influence regeneration ability of plants [14,50]. Generally, it is anticipated that axillary bud culture is the process offering least risk of genetic instability since meristems are more resistant to genetic changes than disorganized plant tissues. Our study reported that, 5 day old seedlings of HCN (half cotyledonary node) showed maximal regeneration frequency as compared to TCN or WCN on combinations of KT + NAA, and KT + NAA + 6-BAP. In soybean cotyledonary node was shown to significantly increased regeneration frequency [51]. On the other hand, WCN showed reduced number of shoots than all HCN treatments, while TCN was the least responsive on all media combinations used. Therefore, we observed that HCN explant was most amenable to establishment of regeneration protocol of white lupin on all kinds of media and phytohormonal combinations [52]. Shivanand et al. [52] reported maximum number of shoot formation from cotyledon explant in *Hisar Lalima*. In addition, our findings showed that shoot regeneration from white lupin cotyledon (HCN) can be induced under normal light condition that may have useful application in gene expression studies involving light responsive genes. Shoot regeneration frequency and number of shoots induced per explant were greatly affected by explant type and preparation of explant (removal of primary leaf or bud from the growing point).

Media components and types effect the shoot regeneration efficiency of plant [53,54,55], and it is important to optimize an appropriate media type and concentration for the establishment of successful regeneration protocol [56]. Several studies showed that, MS media (Murashige and skoog) is most preferably used for in vitro based shoot regeneration and tissue culture purposes. We designed four different ratios of M519 media (M519, ½ M519, ^1/3^ M519, and ¼ M519) to evaluate the effect of different dilutions of media components on regeneration ability of white lupin. Results showed that, ½ M519 media significantly higher regeneration frequency compared to all other media formulations, while M519 and 1/3 M519 showed no obvious differences, suggesting that regeneration response appeared to be largely influenced by genotype selection and media concentration used. Our data indicated that the composition of media and appropriate concentration plays vital role in establishing tissue culture regeneration system in white lupin.

Plant growth regulators play critical role in cell differentiation, division, and elongation [57,58]. Indeed, KT and NAA were found to play an important role in multiple shoot formation [59,60]. We found that, ½ M519 media fortified with KT 4.0 mg L^−1^ + NAA 0.1 mg L^−1^ showed maximum of 80% shoot regeneration frequency by using HCN explants, while all other combinations also induced shoot formation, albeit with a reduced percentage. Additionally, explant types (HCN, TCN, and WCN) exhibited different responses to different combinations of plant hormones (KT, NAA, and 6-BA), suggesting that, regeneration ability of plant depends on explant type and age [22,61].

The efficiency of shoot regeneration is also affected by carbon source which serves as an energy source and helps in maintaining osmotic balance, photomixotrophic metabolism, and promoting plant growth [62,63,64,65]. Sucrose was shown to play a vital role in improving plant growth and shoot regeneration rate, while glucose and maltose played a minor role in shoot induction. The findings in the current study are consistent with previous studies which reported that sucrose induced shoot formation of Soybean [66], Velvet bean [67], Peach [68], Gotu Kola [16], Pea [69,70], Kris [71], Cucumber [72]. The types of carbohydrate sources, and their concentrations may influence culturing success [63,65]. 

Browning is a major constraint to establishment of a successful tissue culture regeneration protocol and it occurs due to oxidation of phenolic compounds. Activated charcoal (AC) was added into the medium to detoxify the effect of phenolic compounds. AC not only increased the number of shoots induced but also improved shoot length. Root induction is highly complex energy requiring process which is based on carbohydrates [68]. Indole acetic acid (IAA) alone and in combination with KT, and IBA played efficient role in promoting root induction and maturation of white lupin. The best concentration optimized for root induction was on ½ M519 medium augmented with IAA 1.0 mg L^−1^ + KT 0.1 mg L^−1^ and IAA 1.0 mg L^−1^ showed 80% and 66% root induction efficiency respectively. 

As a conclusion, we established simple successful regeneration protocol of white lupin by using cotyledonary node, approximately, 8-week duration is required for initial culture to regenerate into whole plant. By using this optimized protocol approximately 40 plants can be obtained from one explant (HCN) within 9 to 10 months. The highest regeneration potential makes this species an ideal candidate for generation of transgenic plant in future. This protocol can be helpful in future for rapid and large scale shoot propagation and genetic improvement of *Lupinus albus* and other crops. The successfully established regeneration protocol makes this species an ideal candidate crop for future genetic improvement and transformation-based studies.

## 4. Material and Methods

### 4.1. Plant Material and Seed Germination 

Mature white lupin (*Lupinus albus L.)* seeds were collected from Joint International Research Laboratory of Water and Nutrient in Cops, Fujian Agriculture and Forestry University, China. Seed surfaces were sterilized by rinsing with 75% ethanol for 1 min, and soaking in 10% NaClO (sodium hypochlorite) solution for 15 minutes. Finally, seeds were washed 3 to 4 times with distilled water to remove any traces of NaClO. The excess water was removed by placing the seeds on sterile filter paper. Sterilized seeds were placed on ½ M519 media in the dark plant growth chamber. After 5days, seed coat was carefully removed and three different explants were prepared from the bases of leaf/bud excision and cotyledon separation: WCN (whole cotyledonary node), HCN (half cotyledonary node), TCN (traditional cotyledonary node). Explant were named on the basis of cotyledon separation and newly emerging bud excision. For example, when cotyledon was separated with the help of scalpel into two halves; one side have some bud while other side was completely clean (without bud), one having bud is WCN, while other is TCN. If cotyledon were excised with the help of blade from the middle in two halves; both side will have some bud these two are named as HCN. 

### 4.2. Culture Medium and Preparation

Murashige and Skoog (M519) basal medium along with salts and vitamins was obtained from Phytotechnology laboratories, United States. Full strength (4.43 g L^−1^) medium was used according to the manufacturer’s protocol along with 3% sucrose, 0.05% MES with different combinations of KT (Kinetin), NAA (Naphthalene acetic acid), and 6-BA (6-Benzyl amino purine) for shoot induction (Table 1), and IAA (Indole acetic acid), IBA (Indole butyric acid), KT (Kinetin) alone or in combination for root induction (Table 2). Medium pH was adjusted to 5.7–5.8, and 6 g L^−1^ agar (Sigma–Aldrich) was added for solidification. Sterilization was done by autoclaving at 121 °C at 15 lbs pressure for 20 min. Phytohormonal stock (KT, NAA,6-BA, IAA, IBA) was sterilized with 0.45 um membrane filter and added into the medium after sterilization. All media and plant hormones stock were placed at 4 °C in a sterilized environment. Total of 40 explants were cultured on four different formulations of M519 medium along with various phytohormonal concentrations and repeated at least four times to improve reliability of the data. 

### 4.3. Initiation of Shoot Induction

Five-day old seedlings were removed from germination medium, and excised from root and hypocotyl. Cotyledon was carefully opened with the help of sterilized scalpels and all primary buds were removed and slightly injured at vertically growing point with sterilized blade, to obtain three explants (HCN, WCN, TCN). Explants were cultured on four different shoot induction media (M519, ½ M519, ^1/3^ M519, ¼ M519), with 3% of four different carbon sources (i.e., sucrose, fructose, glucose, maltose), containing different concentrations and formulations of KT, NAA and BAP, (Table 1). After 2 weeks, explants were transferred to a fresh medium under the same culture conditions to maintain appropriate nutrient supply. Each experiment was repeated three times with 40 cotyledonary node explants per experiment.

### 4.4. Shoot Induction and Elongation 

An in vitro generated sterilized 5 day old seedling was used as a starting material to culture on shoot induction medium. M519 media (M519, ½ M519, ^1/3^ M519, and ¼ M519), augmented with various combinations of KT, NAA, and 6-BAP (Table 1) were used for shoot induction, proliferation, and elongation. After 4 weeks of culturing, the selected in vitro multiple shoot clumps were dissected out and transferred onto the same medium for further growth and development. After two sub-cultures the regenerated shoots were transferred to the same medium along with activated charcoal (0.25 g L^−1^) to prevent browning for elongation. All in vitro cultures were conducted in sterilized air flow cabinet, and placed in controlled plant growth chamber at temperature 24 °C to 25 °C, 60% humidity, and 3000 lux light intensity (white fluorescence light). For each experiment, 40 explants were used and repeated three times for data accuracy. The regeneration efficiency of shoot was calculated by using this formula:

$$\mathrm{Shoot}\;\mathrm{regeneration}\;\mathrm{efficiency}\;\left(\%\right)\;=\frac{\mathrm{Number}\;\mathrm{of}\;\mathrm{shoot}\;\mathrm{induced}\times 100}{\mathrm{Total}\;\mathrm{number}\;\mathrm{of}\;\mathrm{explants}}$$

### 4.5. Root Induction

Elongated shoots >5cm were obtained after 4 sub-cultures, dissected individually without any damage, and placed on M519 medium, augmented with 3% carbon source, 0.05% MES, IAA (Indole acetic acid), IBA (Indole butyric acid), and KT (Kinetin) for root induction (Table 2). Root induction efficiency was calculated by using this formula:

$$\mathrm{Root}\;\mathrm{induction}\;\mathrm{efficiency}\;\left(\%\right)\;=\frac{\mathrm{Number}\;\mathrm{of}\;\mathrm{root}\;\mathrm{induced}\times 100}{\mathrm{Total}\;\mathrm{number}\;\mathrm{of}\;\mathrm{explants}}$$

### 4.6. Statistical Analysis 

Data were recorded on the basis of visual observation of multiple shoot induction, and successful root induction. All data were analyzed by calculating mean ± and standard error using ANOVA (analysis of variance by SAS 9.4 (Statistical Analysis System) software (Cary, North Carolina, U.S). The differences among mean values were determined by Student *t*-test at 5% (*p* > 0.05) significance level.

## Figures and Tables

**Table 1:** Effect of different concentrations of plant growth regulators on in vitro generated shoot multiplication on ½ M519 medium by using HCN as an explant. Different alphabetic letters show significant differences among all combinations used for shoot regeneration in white lupin.

Effect of Different Concentration of Plant Growth Regulators on Shoot Regeneration (mg/L)			Length of Shoot after 14 days of Initiation	Length of Shoot after 21 days of Initiation	Length of Shoot after 28 days of Initiation	No. of Shoot Explant^−1^ (Mean ± SE)	Percentage of Shoot (%)
KT	NAA	BAP					
1.0	0.1	0	2.0 cm	4.0 cm	4.6 cm	9 ± 0.00 ^cd^	45%
1.0	0.2	0	2.4 cm	3.8 cm	4.8 cm	8 ± 0.33 ^de^	40%
1.0	0.4	0	2.2 cm	3.7 cm	4.2 cm	9 ± 0.33 ^cd^	45%
2.0	0.1	0	2.5 cm	3.2 cm	4.0 cm	12 ± 1.15 ^b^	60%
2.0	0.2	0	2.1 cm	3.5 cm	4.5 cm	10 ± 0.44 ^bc^	50%
2.0	0.4	0	2.5 cm	3.4 cm	4.6 cm	11 ± 0.33 ^b^	55%
4.0	0.1	0	3.4 cm	5.0 cm	6.1 cm	16 ± 0.57 ^a^	80%
4.0	0.2	0	3.0 cm	5.5 cm	5.8 cm	9 ± 0.57 ^cd^	45%
4.0	0.4	0	2.9 cm	5.0 cm	5.6 cm	10 ± 0.16 ^bc^	50%
1.0	0.1	1.67	2.0 cm	4.0 cm	4.7 cm	5 ± 0.72 ^f^	25%
2.0	0.1	1.67	1.5 cm	3.0 cm	4.3 cm	3 ± 0.57 ^g^	15%
4.0	0.1	1.67	2.2 cm	3.5 cm	4.2 cm	12 ± 1.15 ^b^	60%

**Table 2:** Effect of different concentrations of Indole acetic acid (IAA), Indole butyric acid (IBA), and Kinetin (KT) on root induction. Different alphabetic letters show significant differences among all combinations used for shoot regeneration in white lupin.

Effect of Different Concentration of Plant Growth Regulators on Shoot Regeneration (mg/L)			No. of Explants Used	No. of Root Induced Explant^−1^ (Mean ± SE)	Percentage of Root Induction
IAA	IBA	KT			
1.0	−	−	15	9.66 ± 0.33 ^bc^	64%
2.0	−	−	15	7.68 ± 0.33 ^d^	51%
3.0	−	−	15	7.00 ± 0.57 ^de^	46%
−	1.0	0.1	15	5.00 ± 0.00 ^g^	33%
−	2.0	0.2	15	7.00 ± 0.57 ^de^	46%
−	3.0	0.3	15	5.33 ± 0.33	33%
1.0	−	0.1	15	12.0 ± 0.57 ^a^	80%
2.0	−	0.2	15	10.0 ± 0.88 ^b^	66%
3.0	−	0.3	15	10.0 ± 0.57 ^b^	66%
1.0	0.5	0	15	6.0 ± 0.33 ^f^	40%
2.0	0.5	0	15	9.0 ± 0.33 ^bc^	60%
3.0	0.5	0	15	7.0 ± 0.33 ^de^	46%

## Abbreviations

KT (kinetin), NAA (naphthalene acetic acid), 6-BAP (6-benzyl amino purine), IAA (indole acetic acid), IBA (indole butyric acid), AC (activated charcoal), M519 (murashige and skoog medium), TCN (traditional cotyledonary node), HCN (half cotyledonary node), WCN (whole cotyledonary node).
